# Supplementary material for: A scaling law to model the effectiveness of identification techniques
Source: Nat Commun. 2025 Jan 9;16:347. doi: 10.1038/s41467-024-55296-6 (PMC11718298; doi:10.1038/s41467-024-55296-6)
Supplement: Supplementary file 1 — Supplementary information [file 41467_2024_55296_MOESM1_ESM.pdf]

Supplementary Information for

A scaling law to model the effectiveness of identification techniques

Luc Rocher,[1,2,3] Julien M. Hendrickx,[2*] Yves-Alexandre de Montjoye[3,4*]

[1] Oxford Internet Institute, University of Oxford, Oxford, OX1 3JS, UK

[2] Information and Communication Technologies, Electronics and Applied Mathematics (ICTEAM), Université catholique de Louvain, B-1348, Louvain-la-Neuve, Belgium

[3] Data Science Institute, Imperial College London, London, SW7 2AZ, UK

[4] Department of Computing, Imperial College London, London, SW7 2AZ, UK

*These authors contributed equally.

Contents

S1 Data description and data availability 3
- S1.1 Tabular data for exact matching experiments . . . 3
- S1.2 Set-valued data for sparse matching experiments . . . 4
- S1.3 Robust matching experiments . . . 4
- S1.4 Columnsets used in figures . . . 4
- S1.5 Data acknowledgments and accessibility . . . 5

S2 Methods 6
- S2.1 Expression for privacy metrics in exact matching attacks . . . 6
- S2.2 Pitman-Yor priors and expected privacy metrics . . . 8
- S2.3 Maximum a-posteriori (MAP) estimates from Pitman-Yor priors . . . 10
- S2.4 Privacy metrics for uniform distributions . . . 11

S3 Supplementary Notes 11
- S3.1 Entropy baseline for exact matching . . . 11
- S3.2 Extrapolation baselines . . . 12

S3.3 Evaluation of the extrapolation methods 13
S3.4 Sensitivity analysis for uniqueness and correctness 13

S4 Supplementary Tables S1 to S8 **13**

S5 Supplementary Figures S1 to S19 **22**

S1 Data description and data availability

S1.1 Tabular data for exact matching experiments

We used, for this project, a rich collection of 250 different datasets from five corpora with various levels of correctness and socio-demographic, survey, and health attributes that would be reasonable quasi-identifiers.

The USA datasets are extracted from the 1-percent Public Use Microdata Sample (PUMS) files, a collection of 3,061,692 individual records from the 2010 US Census of Population and Housing [1]. The PUMS files are available online on the US Census Bureau website and contain 11 attributes we use: state FIP, county, PUMA, number of vehicles, sex, date of birth, marital status, race, educational attainment, employment status, and occupation.

The ADULT datasets are extracted from the Adult Income files, a canonical Machine Learning dataset, composed of 32,561 individuals from the 1994 US Census database. The ADULT files are available online in the UCI Machine Learning Repository [2] and contain 10 nominal and ordinal attributes we use: age, workclass, years of education, marital status, occupation, relationship, race, sex, hours of work per week, and native country.

The HDV datasets are extracted from the Histoire de Vie files, composed of 13,500 individual responses to a 2003 survey from the French National Institute of Statistics and Economic Studies (INSEE), and available on INSEE's website [3]. After pre-processing and removal of null responses, the files contains 632 attributes we use for 8403 individuals.

The MIDUS datasets are extracted from the Midlife in the United States (MIDUS) files, a longitudinal survey of 7,108 individuals comprising physical health, psychological well-being, and social variables. The survey files are available on the Inter-university Consortium for Political and Social Research (ICPSR) website [4]. After pre-processing and removal of null responses, the files contains 415 attributes.

The WEB datasets are extracted from the EFF's Panopticlick study on web fingerprints, collected by the Electronic Frontier Foundation [5, 6]. We use a sample of 5,463,165 records and 8 attributes: javascript enabled, cookie enabled, user agent, http accept, installed plugins, installed fonts, timezone, video resolution, with all cell values replaced by a unique pseudorandom hash.

Finally, we also added 150 synthetic datasets drawn from geometric, Poisson, and Zipf distributions. We used the following parameters for each:

1. $p = 10^{-1.5}$ to $p = 10^{-5}$ (50 parameters, evenly spaced in log-space) for geometric
2. $\mu = 10^{3.0}$ to $\mu = 10^{9.5}$ (50 parameters, evenly spaced in log-space) for Poisson
3. $a = 1 + 10^{-0.2}$ to $a = 1 + 10^{-1.5}$ (50 parameters, evenly spaced in log-space) for Zipf

These parameters are chosen to obtain samples of 10^4 records with a correctness ranging approximately from 0 to 1. For each respective distribution Geom(p), Poisson(μ), and Zipf(a), we draw 10^4 records and consider each sample as one dataset, overall totalling 150 synthetic datasets.

S1.2 Set-valued data for sparse matching experiments

We also use set-valued datasets from three corpora.

The CALLS corpus is a collection of times called where placed or received by users' phones [7]. It originates from an anonymized mobile phone dataset that contains call information for users of a mobile phone operator in a western country. The dataset includes, for every user, the time (rounded to 10min windows) during which they interacted with the mobile phone operator network by initiating or receiving a call. It includes 319,500 unique users.

The APPS corpus is a collection of installed Android apps on 55,278 devices. The dataset includes, for each non-identified phone, a list of hashed identifiers for installed Android apps. It was originally collected by the Carat team at the University of Helsinki, Department of Computer Science [8]. We select a sample of 31,100 users with more than two apps installed each.

The SHOPS corpus is a collection of online shopping orders by 131,200 users. The dataset includes, for each user, a list of hashed identifiers for items bought on the platform. It was originally collected by Instacart and publicly released in 2017 [9].

S1.3 Robust matching experiments

The robust matching experiments include a combination of reported results from four previous studies.

The FACEREC corpus includes measurements of rank-1 identification rates (IR) from the MegaFace benchmark of ML to identify individuals from 2 to 1,000,001 face photographs [10]. It includes only data reported by the authors in their supplementary data files, for all ML methods tested.

The GEO corpus includes measurements of rank-1 IR on mobile phone mobility data from 10 to 500,000 mobile phones [11]. The corpus includes five measurements studies using standard ML methods (L1, Cosine, Bhat, JS, triplet loss) to match mobility data across time by generating a unique profile of participants' mobility patterns.

The IIG corpus includes measurements of rank-1 IR from interactions graphs (1-hop, 2-hop, 3-hop) of mobile phone users, reported by Creţu et al. [12] from 2 to 43,000 users.

The TEXT corpus include measurements of rank-1 IR from written texts reported by Saedi and Dras [13] on 2 to 500 authors in known authorship and one-shot scenarios, using CNN, Siamese Networks, and cosine similarity ML models on a combination of datasets and pre-training scenarios.

S1.4 Columnsets used in figures

In the main text and supplementary figures, our figures display the correctness of a subset of all 400 exact datasets, 58 robust datasets, and 18 sparse datasets. Below are the dataset names and associated columnsets of the datasets used in all figures:

1. Exact:

- USA-1 with columns Age, Geo, Sex
- ADULT-1 with columns Age, Income, Occupation, Relationship, Workclass
- HDV-1 with columns acond3, dd2pos1, dd3pos2, dicaus3, dicaus8, ftnom, ful3mat, glvie, nseul
- WEB-1 with columns UserAgent, Video
- WEB-2 with columns HTTPAccept, CookiesEnabled, JavaScriptEnabled, Timezone, DisplaySize, InstalledFonts, InstalledPlugins, UserAgent, Video
- MIDUS-1 with columns A1PMEDUM, A1SA1S1_14, A1SA32, A1SI29D
- ZIPF-1 with attribute $a = 1.303133$
- GEOM-1 with attribute $p = 1\ 10^{-2}$
- POISSON-1 with attribute $\mu = 10^3$

2. Sparse:

   - APPS-1 with $p = 2$
   - SHOPS-1 with $p = 1$
   - CALLS-1 with $p = 8$

3. Robust:

   - FACEREC-1 with matching using the DeepsenseSmall method
   - FACEREC-2 with matching using the FaceNet method
   - GEO-1 with matching using the Cosine method
   - TEXT-1 with matching using the FF-5K Siam-Cos method
   - IIG-1 with matching using 1-hop social graphs

S1.5 Data acknowledgments and accessibility

We are grateful for data provision to the Electronic Frontier Foundation and in particular William Budington (WEB corpus from the Panopticlick study), N. Asokan and Hien Truong (APPS corpus), as well as Ana-Maria Creţu, Ali Farzanehfar, and Arnaud Tournier. To replicate our experiments, we make available upon request the datasets containing the measurements of correctness, uniqueness, and k-anonymity computed for all exact, sparse, and robust experiments.

S2 Methods

S2.1 Expressions for κ, Ξ, and V_k in exact matching attacks

We study the expected value of privacy metrics, in a gallery $\boldsymbol{G} = \{x_l\}_{l=1}^n$ containing n enrolled records, each drawn i.i.d. from the discrete distribution X. We model any privacy metric $\Psi_{\boldsymbol{G}}$ defined as the mean of an individual metric $\psi_{\boldsymbol{G}}$, over all its records:

$$\Psi_{\boldsymbol{G}} = \frac{1}{n}\sum_{i=1}^{n} \psi_{\boldsymbol{G}}(x_i) \tag{S1}$$

and its expected value:

$$\begin{aligned}
\Psi &= \mathbb{E}_{\boldsymbol{G}\sim X^n}\left[\Psi_{\boldsymbol{G}}\right] && \text{(S2)}\\
&= \mathbb{E}_{\boldsymbol{G}\sim X^n}\left[\frac{1}{n}\sum_{i=1}^{n} \psi_{\boldsymbol{G}}(x_i)\right] && \text{(S3)}\\
&= \mathbb{E}_{\boldsymbol{G}\sim X^n}\left[\psi_{\boldsymbol{G}}(x_1)\right] && \text{(S4)}\\
&= \mathbb{E}_{x\sim X}\left[\psi(x,n)\right] && \text{(S5)}\\
& && \text{(S6)}
\end{aligned}$$

with $\psi(x,n) = \mathbb{E}_{\boldsymbol{G}\sim X^n,\, x\in \boldsymbol{G}}\left[\psi_{\boldsymbol{G}}(x)\right]$.

Uniqueness. Within the gallery $\boldsymbol{G}$, the uniqueness Ξ is the expected fraction of records $x \in \boldsymbol{G}$ with a unique set of auxiliary information $\phi(x)$ [14], with X_ϕ its marginal distribution. We compute its expression using the following expressions:

$$\psi_{\boldsymbol{G}}(x_i) = [\phi(x_i) \text{ unique in } \{\phi(x_i)\}_{i=1}^n] \tag{S7}$$

$$\psi(x,n) = \mathbb{P}(\phi(x) \text{ unique in a sample of } n \text{ records}) \tag{S8}$$

We then derive:

$$\begin{aligned}
\Xi(n) &= \sum_{x\sim X} p(x)\ \mathbb{P}(\phi(x) \text{ unique in a sample of } n \text{ records}) && \text{(S9)}\\
&= \sum_{x_\phi\sim X_\phi} p(x_\phi)\ \mathbb{P}(x_\phi \text{ unique in a sample of } n \text{ records}) && \text{(S10)}\\
&= \sum_{x_\phi\sim X_\phi} p(x_\phi)\ \mathbb{P}(x_\phi \text{ abstent in a sample of } n-1 \text{ records}) && \text{(S11)}\\
&= \sum_{x_\phi\sim X_\phi} p(x_\phi)\ \mathbb{P}(T_{x_\phi,n-1} = 0) && \text{(S12)}
\end{aligned}$$

with $T_{x_\phi,m}$ a random variable counting potential matches for x_ϕ amongst m records. With $p(x_\phi) = \mathbb{P}(X_\phi = x_\phi)$, the variable $T_{x_\phi,m}$ follows a Binomial distribution $B(p(x_\phi), m)$, hence:

$$\Xi(n) = \sum_{x_\phi \sim X_\phi} p(x_\phi) \left(1 - p(x_\phi)\right)^{n-1} \tag{S13}$$

Correctness. Similarly, we can derive the correctness κ, the expected fraction of records $x \in \boldsymbol{G}$ correctly matched from their auxiliary information x_ϕ [14] using

$$\psi(x, n) = \mathbb{P}(x_\phi \text{ correctly matched in a sample of } n \text{ records}) \tag{S14}$$

$$= \sum_{k=1}^{n} \mathbb{P}(k \text{ instances of } x_\phi \text{ in a sample of } n \text{ records})$$

$$\mathbb{P}(\text{correct match amongst } k \text{ candidates}) \tag{S15}$$

$$= \sum_{k=1}^{n} \mathbb{P}(T_{x_\phi,n} = k)\,\frac{1}{k} \tag{S16}$$

$$= \sum_{k=0}^{n-1} \frac{\mathbb{P}(T_{x_\phi,n-1} = k)}{k+1} \tag{S17}$$

hence

$$\kappa(n) = \sum_{x \sim X} p(x)\psi(x, n) \tag{S18}$$

$$= \sum_{x_\phi \sim X_\phi} p(x_\phi)\,\frac{\left[1 - p(x_\phi)\right]^n}{n\,p(x_\phi)} \tag{S19}$$

***k*-anonymity violations.** Finally, the expected fraction of k-anonymity violations V_k is expressed using:

$$\psi(x, n) = \mathbb{P}(1 \text{ to } k-1 \text{ records share } \phi(x) \text{ amongst } n) \tag{S20}$$

$$= \mathbb{P}(1 \le T_{x_\phi,n} \le k-1) \tag{S21}$$

$$= \mathbb{P}(T_{x_\phi,n-1} \le k-2) \tag{S22}$$

$$= I_{1-p(x_\phi)}((n-1) - (k-2), (k-2) + 1) \tag{S23}$$

with I the regularized incomplete beta function. Hence:

$$V_k(n) = \sum_{x_\phi \sim X_\phi} p(x_\phi)\,\mu(x_\phi) \tag{S24}$$

$$= \sum_{x_\phi \sim X_\phi} p(x_\phi)\,I_{1-p(x_\phi)}(n - k + 1, k - 1) \tag{S25}$$

S2.2 Pitman-Yor priors and expected privacy metrics

We denote by $PY(d, \alpha)$ the Pitman-Yor process with a discount parameter $d \in [0, 1]$ and a concentration parameter $\alpha \in [-d, +\infty]$. Distributions $\pi \sim PY(d, \alpha)$ can be drawn using a generative "stick-breaking" process, by sampling independent Beta random variables:

$$\beta_i \sim \text{Beta}(1 - d, \alpha + i\, d), \quad \pi_i = \prod_{j=1}^{i-1}(1 - \beta_j)\beta_i \tag{S26}$$

with π_i the probability associated with the i-th class in the population.

Pitman and Yor proved in 1997 the following proposition:

Proposition 1. *For $Y \sim PY(d, \alpha)$, a countably-infinite distribution $(y_i)_{1 \leq i \leq +\infty}$, and $f : [0, 1] \rightarrow \mathbb{R}$ a continuous function:*

$$\mathbb{E}_{(Y \mid d, \alpha)}\left[\sum_{i=1}^{+\infty} f(y_i)\right] = \mathbb{E}_{(\tilde{\pi}_1 \mid d, \alpha)}\left[\frac{f(\tilde{\pi}_1)}{\tilde{\pi}_1}\right] \tag{S27}$$

with $\tilde{\pi}_1 \sim Beta(1 - d, d + \alpha)$ the first size-biased sample from Y.

This allows us to obtain close-form expressions for the expected entropy, uniqueness, correctness, and k-anonymity violations.

Proposition 2. *For $Y \sim PY(d, \alpha)$, the expected entropy is:*

$$\mathbb{E}\left[\mathbb{H}(Y) \mid d, \alpha\right] = \psi_0(\alpha + 1) - \psi_0(1 - d) \tag{S28}$$

Proof. See the proof by Archer *et al.* [15] using $f(\pi) = \pi \log_2 \pi$. □

Proposition 3. *For $Y \sim PY(d, \alpha)$, the expected uniqueness in a gallery of n records is:*

$$\mathbb{E}\left[\Xi(n) \mid d, \alpha\right] = \frac{\Gamma(\alpha + 1)}{\Gamma(d + \alpha)} \frac{\Gamma(n + d + \alpha - 1)}{\Gamma(n + \alpha)} \tag{S29}$$

Proof. We use the Proposition 1 with $f(\pi) = \pi\,(1 - \pi)^{n-1}$:

$$\begin{aligned}
\mathbb{E}\left[\Xi(n) \mid d, \alpha\right] &= \frac{1}{\mathrm{B}(1 - d, d + \alpha)} \int_0^1 \frac{f(\pi)}{\pi} \pi^{-d} (1 - \pi)^{d + \alpha - 1} \,\mathrm{d}\pi \\
&= \frac{1}{\mathrm{B}(1 - d, d + \alpha)} \int_0^1 \pi^{-d} (1 - \pi)^{n + d + \alpha - 2} \,\mathrm{d}\pi \\
&= \frac{1}{\mathrm{B}(1 - d, d + \alpha)} \mathrm{B}(1 - d, n + d + \alpha - 1) \\
&= \frac{\Gamma(1 + \alpha)\,\Gamma(n + d + \alpha - 1)}{\Gamma(d + \alpha)\,\Gamma(n + \alpha)}
\end{aligned} \tag{S30}$$

□

Proposition 4. *For $Y \sim PY(d, \alpha)$, the expected correctness in a gallery of n records is:*

$$\mathbb{E}\left[\kappa \mid d, \alpha\right] = \frac{1}{nd}\left(\frac{\Gamma(1+\alpha)}{\Gamma(d+\alpha)}\frac{\Gamma(n+d+\alpha)}{\Gamma(n+\alpha)} - \alpha\right) \tag{S31}$$

Proof. We use the Proposition 1 with:

$$\begin{aligned} f(\pi) &= \pi \, \frac{1}{n\,\pi}\left(1-(1-\pi)^n\right) \\ &= \frac{1}{n}\left(1-(1-\pi)^n\right) \end{aligned} \tag{S32}$$

so that:

$$\begin{aligned} \mathbb{E}\left[\kappa(n) \mid d, \alpha\right] &= \frac{1}{\mathrm{B}(1-d, d+\alpha)} \int_0^1 \frac{f(\pi)}{\pi} \pi^{-d}(1-\pi)^{d+\alpha-1}\,\mathrm{d}\pi \\ &= \frac{1}{\mathrm{B}(1-d, d+\alpha)} \int_0^1 \frac{1}{\pi}\frac{1}{n}\left(1-(1-\pi)^n\right)\pi^{-d}(1-\pi)^{d+\alpha-1}\,\mathrm{d}\pi \\ &= \frac{1}{n}\frac{1}{\mathrm{B}(1-d, d+\alpha)} \int_0^1 \pi^{-d-1}(1-\pi)^{\alpha+d-1} - \pi^{-d-1}(1-\pi)^{n+\alpha+d-1}\,\mathrm{d}\pi \\ &= \frac{1}{n}\frac{1}{\mathrm{B}(1-d, d+\alpha)}\left[\mathrm{B}(-d, d+\alpha) - \mathrm{B}(-d, n+\alpha+d)\right] \\ &= \frac{1}{nd}\left(\frac{\Gamma(1+\alpha)}{\Gamma(d+\alpha)}\frac{\Gamma(n+d+\alpha)}{\Gamma(n+\alpha)} - \alpha\right) \end{aligned} \tag{S33}$$

□

Proposition 5. *For $Y \sim PY(d, \alpha)$, the expected fraction of k-anonymity violations in a gallery of n records is:*

$$\begin{aligned} \mathbb{E}\left[V_k(n) \mid d, \alpha\right] &= \int_0^1 \frac{I_{1-\pi}(n-k+1, k-1)}{B(1-d, d+\alpha)} \pi^{-d-1}(1-\pi)^{d+\alpha-1}\,d\pi \\ &= \binom{n-1}{n-k+1}\Gamma(k-d)\,\Gamma(n-k+1+d+\alpha) \\ &\quad \frac{{}_3F_2(1, n, n-k+1+d+\alpha; n-k+2, n+1+\alpha, 1)}{\mathrm{B}(1-d, d+\alpha)\,\Gamma(n+1+\alpha)} \end{aligned} \tag{S34}$$

Proof. We use the Proposition 1 with $f(\pi) = \pi\, I_{1-\pi}(n-k+1, k-1)$ to obtain:

$$\begin{aligned} \mathbb{E}\left[V_k(n) \mid d, \alpha\right] &= \frac{1}{\mathrm{B}(1-d, d+\alpha)} \int_0^1 \frac{f(\pi)}{\pi} \pi^{-d}(1-\pi)^{d+\alpha-1}\,\mathrm{d}\pi \\ &= \frac{1}{\mathrm{B}(1-d, d+\alpha)} \int_0^1 I_{1-\pi}(n-k+1, k-1)\pi^{-d-1}(1-\pi)^{d+\alpha-1}\,\mathrm{d}\pi \end{aligned} \tag{S35}$$

Since $I_z(m,n) = (1-z)^n \sum_{j=m}^{\infty} z^j \binom{j+n-1}{j}$, we obtain:

$$\begin{aligned}\mathbb{E}\left[V_k(n) \mid d,\alpha\right] &= \frac{1}{\mathrm{B}(1-d,d+\alpha)} \sum_{j=n-k+1}^{\infty} \frac{\binom{(k-1)+j-1}{j}\,\Gamma(k-d)\,\Gamma(d+j+\alpha)}{\Gamma(j+k+\alpha)} \\ &= \binom{n-1}{n-k+1}\Gamma(k-d)\,\Gamma(n-k+1+d+\alpha) \\ &\quad \frac{{}_3F_2(1,n,n-k+1+d+\alpha;n-k+2,n+1+\alpha,1)}{\mathrm{B}(1-d,d+\alpha)\,\Gamma(n+1+\alpha)}\end{aligned} \tag{S36}$$

□

S2.3 Maximum a-posteriori (MAP) estimates from Pitman-Yor priors

Given a sample $\boldsymbol{y} = (y_i)_{1\le i\le n}$ from an unknown distribution $Y \sim PY(d,\alpha)$, we have:

$$p(\boldsymbol{y} \mid d,\alpha) = \frac{\prod_{l=1}^{k-1}(\alpha+ld)\ \prod_{i=1}^{K}\Gamma(n_i-d)\ \Gamma(1+\alpha)}{\Gamma(1-d)^K\ \Gamma(\alpha+n)} \tag{S37}$$

with $\boldsymbol{n} = (n_i)_{1\le i\le K}$ the observed frequency counts in $\boldsymbol{y}$ summing to n, and K the total number of frequency classes.

Numerically, computations for $\prod_{l=1}^{k-1}(\alpha+ld)$ can be accelerated by noting that:

$$\begin{aligned}\prod_{l=1}^{k-1}(\alpha+ld) &= d^{K-1}\prod_{l=1}^{k-1}(\alpha/d+l) \\ &= d^{K-1}\frac{\Gamma(\alpha/d+K-1)}{\Gamma(\alpha/d+1)}\end{aligned} \tag{S38}$$

Recall that a PYP is equally parametrized by (d,α) and (h,γ). We place a uniform prior on h and set an exponential prior $q(\gamma)$ on γ:

$$q(\gamma) = \exp(-\frac{10}{1-\gamma}) \tag{S39}$$

We refer to Archer et al. [15] for a detailed study of the priors for PYP distributions.

S2.4 Privacy metrics for uniform distributions

Let Y be a uniform distribution over the d distinct outcomes $\{1, 2, \ldots, d\}$. The expected entropy of Y is $h = \log_2 d$. In a gallery of n records, the expected correctness is

$$\begin{aligned}
\mathbb{E}[\kappa(n)] &= \sum_{i=1}^{d} \mathbb{P}(Y=i) \frac{1}{n\ \mathbb{P}(Y=i)} \left(1 - (1 - \mathbb{P}(Y=i))^n\right) \\
&= \frac{d}{n}\left(1 - \left(1 - \frac{1}{d}\right)^n\right) \\
&= \frac{2^h}{n}\left(1 - \left(1 - 2^{-h}\right)^n\right)
\end{aligned} \tag{S40}$$

and the expected uniqueness is:

$$\begin{aligned}
\mathbb{E}[\Xi(n)] &= \sum_{i=1}^{d} \mathbb{P}(Y=i)\,(1 - \mathbb{P}(Y=i))^{n-1} \\
&= \sum_{i=1}^{d} \frac{1}{d}\left(1 - \frac{1}{d}\right)^{n-1} \\
&= \left(1 - \frac{1}{d}\right)^{n-1} \\
&= \left(1 - 2^{-h}\right)^{n-1}
\end{aligned} \tag{S41}$$

S3 Supplementary Notes

S3.1 Entropy baseline for exact matching

We compare the PYC model with an entropy-based method, assuming a uniform discrete distribution for X_ϕ. Using the equations from Section S2.4, we compute the correctness

$$\kappa_E(n) = \frac{2^{h_E}}{n}\left[1 - \left(1 - 2^{-h_E}\right)^n\right] \tag{S42}$$

with n the population size and h_E the estimated Shannon entropy.

In our experiments, we compare the performance of the PYC and the entropy baseline given access to the same data sample. In the first case, we estimate the MAP parameters h^* and γ^* by fitting the model to the empirical distribution function. In the second case, we estimate the entropy h_E from the empirical distribution function and compute the associated correctness κ_E. Given that there is no unbiased estimator for entropy [16], we use the standard maximum likelihood estimator for the entropy baseline: $\mathbb{H}_{\text{MLE}}(\pi) = -\sum_{i=1}^{m} \pi_i \log_2(\pi_i)$.

S3.2 Extrapolation baselines

We compare our fitted PYC model (PYC-MB) to four baselines, used to extrapolate the correctness $\kappa(n)$ from $t \geq 2$ observations $n_{1:t} = (n^{(1)}, n^{(2)}, \ldots, n^{(t)})$ and $\kappa_{1:t} = (\kappa^{(1)}, \kappa^{(2)}, \ldots, \kappa^{(t)})$. We use 50 data points when available, and less if $t \leq 50$ (e.g., at small sampling fractions for exact and sparse matching) or if the reported measurement data contains less than 50 points.

Entropy baseline (ENT). This baseline follows the model from Section S3.1:

$$\kappa_{\mathrm{ENT}}(n) = \frac{2^h}{n}\left(1 - \left(1 - 2^{-h}\right)^n\right) \tag{S43}$$

with a single parameter h.

Random baseline (RND). We propose a random baseline, assuming only a monotonous decrease of the correctness as n increases. For $n > n^{(t)}$, this baseline therefore returns a fixed correctness uniformly drawn between 0 and $\kappa^{(t)}$. When reporting the RMSE between empirical and expected correctness, we calculate the expected RMSE for $\kappa_{\mathrm{RND}}(n) \sim \mathrm{U}(0, \widehat{\kappa}(n^{(t)}))$:

$$\mathbb{E}\left[\mathrm{RMSE}(\widehat{\kappa}(n), \kappa_{\mathrm{RND}}(n)\right] = \sqrt{\frac{(\widehat{\kappa}(n^{(t)}))^3}{3} + \widehat{\kappa}(n^{(t)})(\widehat{\kappa}(n))^2 - (\widehat{\kappa}(n^{(t)}))^2\widehat{\kappa}(n)} \tag{S44}$$

Exponential decay baseline (EXP). This baseline is an exponential decay model that assumes a functional form

$$\kappa_{\mathrm{EXP}}(n) = a\, e^{-b\,n} + c \tag{S45}$$

with three parameters a, b, c and the constraint $\kappa_{\mathrm{EXP}}(1) = 1$ (2 degrees of freedom). This functional form has been used by Achara et al. [17] to extrapolate the unicity of installed smartphone apps and by Sekara et al. [18] for human mobility traces. It is also used for the NIST's Face Recognition Vendor Test (FRVT) to forecast the accuracy of facial recognition technologies [19].

Polynomial baseline (POL). This baseline is a polynomial model that assumes a functional form

$$\kappa_{\mathrm{POL}}(n) = a\ \log(n)^2 + b\ \log(n) + c \tag{S46}$$

with three parameters a, b, c and the constraint $\kappa_{\mathrm{POL}}(1) = 1$ (2 degrees of freedom). This functional form has been used by Friedman et al. [20] and Baveja et al. [21] to extrapolate the rank-1 identification rate (IR) for facial recognition.

S3.3 Evaluation of the extrapolation methods

We report the performances of the PYC-MB functional form, as well as the four baselines, in Tables S4 and S5 (exact matching), Table S6 (sparse matching), and Tables S7 and S8 (robust matching), with six sampling fractions $\mu = n^{(t)}/n$ of 0.1%, 0.5%, 1%, 5%, 10%, and 100%. A low RMSE on the 100% sampling fraction will indicate a good model specification while a low RMSE on, e.g., the 5% and 10% sampling fractions indicates a good predicting power. Our results show that all baselines perform significantly worse than the PYC-MB model, across identification scenario, corpus, and sampling fraction but one (0.1% for sparse matching).

For all extrapolations experiments (reported in the summary tables and figures S1 to S18), we select only datasets where sample correctness $\widehat{\kappa}(n^{(t)})$, the measurement with the largest population size $n^{(t)}$, is between 0.01 and 0.99. This excludes cases were the sampling fraction is too large or too small for an extrapolation to be meaningful. The column c in Tables S4 to S8 reports the number of selected datasets from each corpus.

S3.4 Sensitivity analysis for uniqueness and correctness

We report the statistical bias $\kappa - \mathbb{E}[\kappa]$ and $\Xi - \mathbb{E}[\Xi]$ on exact matching experiments.

Fig. S1 shows a strong association between statistical bias on the correctness κ and the uniqueness Ξ. This association is expected since the two metrics are positively correlated: the higher the correctness, the higher the uniqueness. This suggests that accurately predicting the correctness implies accurately predicting the uniqueness, and vice versa.

S4 Supplementary Tables S1 to S8

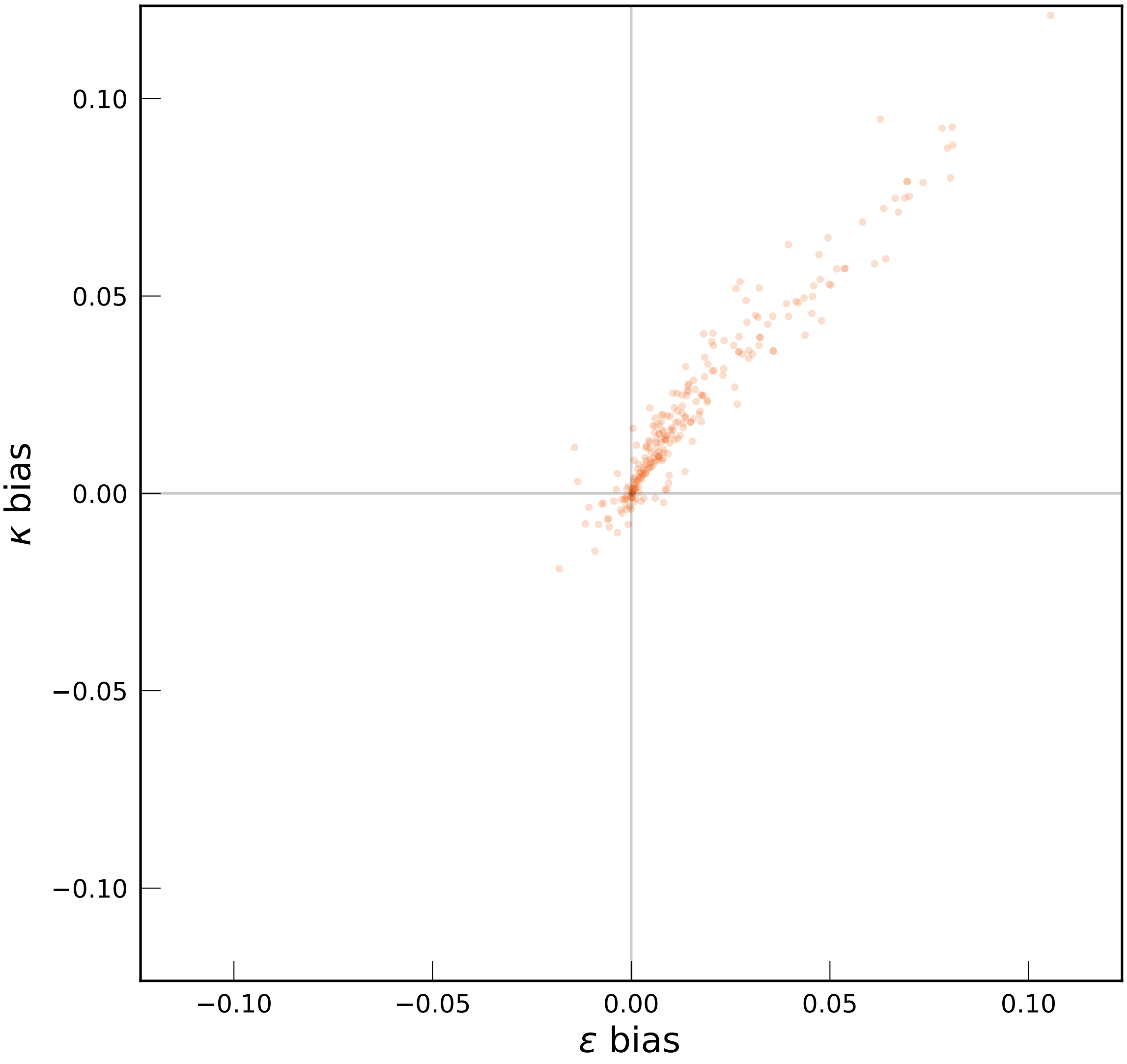

Figure S1: Statistical bias between empirical and predicted values of uniqueness and correctness. We report the difference between empirical values and predicted according to the PYC model, fitted on the frequencies of anonymity sets for exact matching.

Corpus	n	c	PYC RMSE[κ]	PYC RMSE[Ξ]	Entropy baseline RMSE[κ]	Entropy baseline RMSE[Ξ]	PYC RMSE[V_4]	PYC RMSE[V_8]
ADULT	32,561	50	**0.011**	**0.007**	0.223	0.201	0.019	0.024
HDV	8,403	50	**0.023**	**0.014**	0.114	0.115	0.037	0.055
MIDUS	7,108	50	**0.019**	**0.014**	0.232	0.187	0.034	0.037
WEB	5,463,165	50	**0.046**	**0.040**	0.363	0.292	0.062	0.054
USA	3,061,692	50	**0.045**	**0.039**	0.426	0.177	0.066	0.085
Geom	10,000	50	**0.002**	**0.015**	0.158	0.065	0.021	0.043
Poisson	10,000	50	**0.003**	**0.027**	0.169	0.064	0.040	0.082
Zipf	10,000	50	**0.028**	**0.038**	0.268	0.250	0.101	0.090
Overall		400	**0.017**	**0.018**	0.187	0.146	0.041	0.043

Table S1: Empirical validation of the PYC model. We report the results for all selected datasets, grouped by corpus, of the PYC model when estimating κ, Ξ, V_4, V_8, as well as the uniform entropy baseline when estimating κ and Ξ. We also report the population size n and the number of datasets c selected from each corpus. The k-anonymity violation metrics V_4 and V_8 are estimated using numerical integration. In each cell, we report the root mean square error (RMSE) between the empirical and estimated metric, averaged per corpus. We indicate in bold the method with the lowest error rate between PYC and Entropy baseline, for κ and for Ξ.

Corpus	n	c	h^*	$D_{\mathrm{KL}}(\widehat{\pi} \mid\mid \pi)$
ADULT	32,561	50	10.00 ± 2.35	1.14 ± 1.06
HDV	8,403	50	6.96 ± 1.12	0.31 ± 0.26
MIDUS	7,108	50	10.57 ± 5.01	2.38 ± 3.75
WEB	5,463,165	50	20.69 ± 7.00	5.70 ± 6.31
USA	3,061,692	50	22.52 ± 11.68	12.39 ± 14.98
Geom	10,000	50	8.84 ± 3.27	2.37 ± 3.91
Poisson	10,000	50	8.92 ± 3.19	2.63 ± 3.07
Zipf	10,000	50	9.77 ± 4.03	6.23 ± 6.72
Overall		400	12.28 ± 7.87	4.14 ± 7.55

Table S2: Kullback–Leibler divergence between empirical frequencies and distributions fitted by the PYC. We report the KL divergence between the empirical frequencies $\widehat{\pi}$ and the fitted frequencies π obtained by sampling from the Pitman-Yor processes fitted using the MAP parameters h^* and γ^*, for all selected datasets, grouped by corpus. We also report the entropy $h^* = \mathbb{E}[H(\pi)]$ according the fitted Pitman-Yor process, the population size n and the number of datasets c selected from each corpus.

μ	Method	c	RMSE PYC-MB	ENT	EXP	POL	RND
0.1%	Exact	216	**0.290**	0.370	0.315	0.484	0.347
	Sparse	13	0.326	0.484	**0.302**	0.395	0.345
	Robust	30	**0.234**	0.523	0.340	0.386	0.287
0.5%	Exact	290	**0.153**	0.364	0.287	0.355	0.305
	Sparse	16	**0.191**	0.430	0.330	0.369	0.308
	Robust	44	**0.154**	0.479	0.346	0.312	0.287
1%	Exact	316	**0.122**	0.357	0.262	0.330	0.282
	Sparse	16	**0.104**	0.422	0.253	0.393	0.287
	Robust	47	**0.183**	0.463	0.310	0.433	0.254
5%	Exact	351	**0.067**	0.310	0.192	0.179	0.230
	Sparse	17	**0.056**	0.373	0.149	0.149	0.233
	Robust	58	**0.134**	0.411	0.224	0.361	0.216
10%	Exact	365	**0.051**	0.276	0.155	0.146	0.214
	Sparse	17	**0.036**	0.340	0.124	0.092	0.215
	Robust	58	**0.102**	0.379	0.175	0.262	0.191
100%	Exact	400	**0.013**	0.128	0.037	0.028	0.213
	Sparse	18	**0.013**	0.152	0.051	0.055	0.202
	Robust	58	**0.060**	0.256	0.099	0.155	0.172

Table S3: RMSE when extrapolating the correctness κ of exact, sparse, and robust matching. We report the results for all selected data collections, grouped by method, for all sampling fractions μ in 0.1%, 0.5%, 1%, 5%, 10%, 100%. For each data collection, we measure the empirical correctness from $n^{(0)} = 1$ to $n^{(t)} = n\mu$ records, fit four functional forms, and report the mean RMSE between empirical and estimated correctness of n records. The four functional forms are PYC-MB (Pitman-Yor Correctness functional form, 2 degrees of freedom), ENT (Entropy baseline with no tail complexity, 1 d.o.f), EXP (exponential decay function, 2 d.o.f), POL (polynomial function, 2 d.o.f.). We report results for RND (random) where the value for κ is draw uniformly between 0 and $n^{(t)}$. We also report the number of data collections c included from each corpus, left after selecting only data collections for which $0.01 < n^{(t)} < 0.99$. Tables S3-5 show the detailed results per corpus. We indicate in bold the method with the lowest error rate.

μ	Corpus	n	$n^{(t)}$	c	RMSE				
					PYC-MB	ENT	EXP	POL	RND
0.1%	ADULT	32,561	32	27	0.336	**0.257**	0.322	0.603	0.355
	HDV	8,403	8	36	0.378	**0.189**	0.331	0.548	0.393
	MIDUS	7,108	7	29	0.369	0.366	0.340	**0.337**	0.339
	WEB	5,463,165	5,463	37	**0.123**	0.558	0.295	0.378	0.286
	USA	3,061,692	3,061	21	**0.201**	0.346	0.256	0.655	0.355
	GEOM	10,000	10	11	0.264	**0.027**	0.364	0.669	0.513
	POISSON	10,000	10	9	0.306	**0.014**	0.481	0.787	0.527
	ZIPF	10,000	10	46	0.258	0.433	0.263	**0.202**	0.281
	Overall			216	**0.290**	0.370	0.315	0.484	0.347
0.5%	ADULT	32,561	162	38	**0.168**	0.276	0.232	0.369	0.297
	HDV	8,403	42	50	**0.199**	0.215	0.250	0.368	0.316
	MIDUS	7,108	35	40	0.240	0.386	**0.216**	0.314	0.289
	WEB	5,463,165	27,315	41	**0.098**	0.558	0.240	0.240	0.282
	USA	3,061,692	15,308	27	**0.104**	0.377	0.208	0.242	0.308
	GEOM	10,000	50	23	0.103	**0.059**	0.353	0.544	0.409
	POISSON	10,000	50	21	0.130	**0.030**	0.415	0.624	0.438
	ZIPF	10,000	50	50	**0.051**	0.462	0.369	0.189	0.225
	Overall			290	**0.153**	0.364	0.287	0.355	0.305
1%	ADULT	32,561	325	44	**0.146**	0.281	0.211	0.185	0.270
	HDV	8,403	84	50	**0.149**	0.214	0.236	0.338	0.275
	MIDUS	7,108	71	46	**0.169**	0.393	0.207	0.293	0.283
	WEB	5,463,165	54,631	44	**0.105**	0.543	0.210	0.194	0.288
	USA	3,061,692	30,616	28	**0.067**	0.377	0.238	0.161	0.284
	GEOM	10,000	100	28	0.131	**0.063**	0.323	0.479	0.347
	POISSON	10,000	100	26	0.091	**0.035**	0.373	0.556	0.378
	ZIPF	10,000	100	50	**0.038**	0.456	0.316	0.358	0.207
	Overall			316	**0.122**	0.357	0.262	0.330	0.282

Table S4: RMSE when extrapolating the correctness κ of exact matching. We report the results for all selected data collections, grouped by corpus, for three sampling fractions $\mu \in [0.1\%, 0.5\%, 1\%]$. For each data collection, we measure the empirical correctness from $n^{(0)} = 1$ to $n^{(t)} = n\mu$ records, fit four functional forms, and report the RMSE between empirical and estimated correctness of n records. The four functional forms include: PYC-MB (Pitman-Yor Correctness functional form, 2 degrees of freedom), ENT (Entropy baseline with no tail complexity, 1 d.o.f), EXP (exponential decay function, 2 d.o.f), POL (polynomial baseline, 2 d.o.f.). We also report results for RND (expected random baseline between 0 and $n^{(t)}$). We report the population size n, the sample size $n^{(t)}$, and the number of data collections c selected from each corpus. We indicate in bold the method with the lowest error rate.

μ	Corpus	n	$n^{(t)}$	c	RMSE				
					PYC-MB	ENT	EXP	POL	RND
5%	ADULT	32,561	1,628	50	**0.038**	0.252	0.200	0.228	0.222
	HDV	8,403	420	50	**0.087**	0.199	0.191	0.159	0.170
	MIDUS	7,108	355	49	**0.094**	0.342	0.182	0.153	0.245
	WEB	5,463,165	273,158	50	**0.080**	0.454	0.139	0.113	0.301
	USA	3,061,692	153,084	29	**0.049**	0.338	0.270	0.124	0.219
	GEOM	10,000	500	37	0.058	**0.051**	0.208	0.212	0.243
	POISSON	10,000	500	36	0.070	**0.028**	0.236	0.255	0.265
	ZIPF	10,000	500	50	**0.015**	0.424	0.125	0.155	0.182
	Overall			351	**0.067**	0.310	0.192	0.179	0.230
10%	ADULT	32,561	3,256	50	**0.027**	0.234	0.184	0.171	0.197
	HDV	8,403	840	50	**0.070**	0.186	0.157	0.131	0.124
	MIDUS	7,108	710	50	**0.077**	0.297	0.155	0.122	0.234
	WEB	5,463,165	546,316	50	**0.070**	0.406	0.118	0.118	0.299
	USA	3,061,692	306,169	34	**0.041**	0.287	0.177	0.119	0.236
	GEOM	10,000	1,000	41	**0.027**	0.047	0.161	0.194	0.222
	POISSON	10,000	1,000	40	0.036	**0.026**	0.179	0.204	0.237
	ZIPF	10,000	1,000	50	**0.010**	0.394	0.108	0.086	0.177
	Overall			365	**0.051**	0.276	0.155	0.146	0.214
100%	ADULT	32,561	32,561	50	**0.009**	0.133	0.021	0.025	0.170
	HDV	8,403	8,403	50	**0.020**	0.123	0.030	0.025	0.073
	MIDUS	7,108	7,108	50	0.017	0.135	0.022	**0.017**	0.217
	WEB	5,463,165	5,463,165	50	**0.018**	0.177	0.057	0.026	0.296
	USA	3,061,692	3,061,692	50	**0.015**	0.107	0.017	0.037	0.315
	GEOM	10,000	10,000	50	**0.004**	0.027	0.040	0.034	0.224
	POISSON	10,000	10,000	50	**0.004**	0.015	0.047	0.038	0.233
	ZIPF	10,000	10,000	50	**0.002**	0.189	0.042	0.008	0.174
	Overall			400	**0.013**	0.128	0.037	0.028	0.213

Table S5: RMSE when extrapolating the correctness κ of exact matching. We report the results for all selected data collections, grouped by corpus, for three sampling fractions $\mu \in [5\%, 10\%, 100\%]$. For each data collection, we measure the empirical correctness from $n^{(0)} = 1$ to $n^{(t)} = n\mu$ records, fit four functional forms, and then report the RMSE between empirical and estimated correctness of n records. The four functional forms include: PYC-MB (2 degrees of freedom), ENT (Entropy baseline with no tail complexity, 1 d.o.f), EXP (exponential decay function, 2 d.o.f), POL (polynomial baseline, 2 d.o.f.). We also report results for RND (expected random baseline between 0 and $n^{(t)}$). We report the population size n, the sample size $n^{(t)}$, and the number of data collections c selected from each corpus. We indicate in bold the method with the lowest error rate.

μ	Corpus	n	$n^{(t)}$	c	RMSE				
					PYC-MB	ENT	EXP	POL	RND
0.1%	APPS	55,278	55	9	0.314	0.558	0.328	**0.238**	0.310
	CALLS	319,507	319	2	0.480	**0.021**	0.311	0.801	0.519
	SHOPS	131,208	131	2	0.134	0.347	**0.119**	0.340	0.329
	Overall			13	0.326	0.484	**0.302**	0.395	0.345
0.5%	APPS	55,278	276	9	**0.085**	0.546	0.330	0.184	0.282
	CALLS	319,507	1,597	5	0.314	**0.094**	0.388	0.612	0.382
	SHOPS	131,208	656	2	0.112	0.340	0.083	**0.032**	0.242
	Overall			16	**0.191**	0.430	0.330	0.369	0.308
1%	APPS	55,278	552	9	**0.082**	0.537	0.212	0.109	0.270
	CALLS	319,507	3,195	5	0.124	**0.088**	0.341	0.664	0.349
	SHOPS	131,208	1,312	2	**0.135**	0.333	0.140	0.287	0.207
	Overall			16	**0.104**	0.422	0.253	0.393	0.287
5%	APPS	55,278	2,763	9	**0.045**	0.481	0.128	0.128	0.247
	CALLS	319,507	15,975	5	**0.065**	0.080	0.173	0.176	0.193
	SHOPS	131,208	6,560	3	**0.068**	0.289	0.166	0.157	0.255
	Overall			17	**0.056**	0.373	0.149	0.149	0.233
10%	APPS	55,278	5,527	9	**0.038**	0.439	0.114	0.080	0.243
	CALLS	319,507	31,950	5	**0.014**	0.077	0.130	0.129	0.148
	SHOPS	131,208	13,120	3	0.052	0.257	0.140	**0.038**	0.244
	Overall			17	**0.036**	0.340	0.124	0.092	0.215
100%	APPS	55,278	55,278	9	0.015	0.202	0.045	**0.014**	0.238
	CALLS	319,507	319,507	5	**0.007**	0.049	0.073	0.102	0.046
	SHOPS	131,208	131,208	4	0.014	0.098	0.022	**0.010**	0.315
	Overall			18	**0.013**	0.152	0.051	0.055	0.202

Table S6: RMSE when extrapolating the correctness κ of sparse matching. We report the results for all selected data collections, grouped by corpus, for three sampling fractions $\mu \in [0.1\%, 0.5\%, 1\%, 5\%, 10\%, 100\%]$. For each data collection, we measure the empirical correctness from $n^{(0)} = 1$ to $n^{(t)} = n\mu$ records, fit four functional forms, and then report the RMSE between empirical and estimated correctness of n records. The four functional forms include: PYC-MB (Pitman-Yor Correctness functional form, 2 degrees of freedom), ENT (Entropy baseline with no tail complexity, 1 d.o.f), EXP (exponential decay function, 2 d.o.f), POL (polynomial baseline, 2 d.o.f.). We also report results for RND (expected random baseline between 0 and $n^{(t)}$). We report the population size n, the sample size $n^{(t)}$, and the number of data collections c selected from each corpus. We indicate in bold the method with the lowest error rate.

μ	Corpus	n	$n^{(t)}$	c	RMSE				
					PYC-MB	ENT	EXP	POL	RND
0.1%	FACEREC	1,000,001	1,000	26	**0.209**	0.533	0.361	0.396	0.287
	IIG	43,000	43	3	0.320	0.438	**0.177**	0.344	0.288
	GEO	500,000	500	1	0.449	0.498	**0.041**	0.199	0.283
	Overall			30	**0.234**	0.523	0.340	0.386	0.287
0.5%	FACEREC	1,000,001	5,000	26	**0.180**	0.529	0.312	0.335	0.266
	IIG	43,000	215	3	**0.145**	0.431	0.380	0.325	0.250
	GEO	500,000	2,500	5	**0.052**	0.590	0.281	0.300	0.319
	TEXT	500	2	10	**0.111**	0.223	0.436	0.245	0.339
	Overall			44	**0.154**	0.479	0.346	0.312	0.287
1%	FACEREC	1,000,001	10,000	26	**0.164**	0.527	0.287	0.276	0.256
	IIG	43,000	430	3	0.128	0.424	0.318	**0.127**	0.228
	GEO	500,000	5,000	5	**0.065**	0.575	0.306	0.277	0.314
	TEXT	500	5	13	0.247	**0.224**	0.350	0.700	0.234
	Overall			47	**0.183**	0.463	0.310	0.433	0.254

Table S7: RMSE when extrapolating the correctness κ of robust matching. We report the results for all selected data collections, grouped by corpus, for three sampling fractions $\mu \in [0.1\%, 0.5\%, 1\%]$. For each data collection, we measure the empirical correctness from $n^{(0)} = 1$ to $n^{(t)} = n\mu$ records, fit four functional forms, and then report the RMSE between empirical and estimated correctness of n records. The four functional forms include: PYC-MB (Pitman-Yor Correctness functional form, 2 degrees of freedom), ENT (Entropy baseline with no tail complexity, 1 d.o.f), EXP (exponential decay function, 2 d.o.f), POL (polynomial baseline, 2 d.o.f.). We also report results for RND (expected random baseline between 0 and $n^{(t)}$). We report the population size n, the sample size $n^{(t)}$, and the number of data collections c selected from each corpus. We indicate in bold the method with the lowest error rate.

μ	Corpus	n	$n^{(t)}$	c	RMSE PYC-MB	ENT	EXP	POL	RND
5%	FACEREC	1,000,001	50,000	26	**0.126**	0.512	0.219	0.129	0.235
	IIG	43,000	2,150	3	**0.082**	0.389	0.126	0.188	0.187
	GEO	500,000	25,000	5	**0.036**	0.502	0.197	0.170	0.305
	TEXT	500	25	24	**0.158**	0.231	0.243	0.535	0.181
	Overall			58	**0.134**	0.411	0.224	0.361	0.216
10%	FACEREC	1,000,001	100,000	26	**0.108**	0.479	0.186	0.115	0.227
	IIG	43,000	4,300	3	**0.060**	0.361	0.102	0.098	0.176
	GEO	500,000	50,000	5	**0.027**	0.444	0.083	0.072	0.302
	TEXT	500	50	24	**0.108**	0.205	0.184	0.386	0.130
	Overall			58	**0.102**	0.379	0.175	0.262	0.191
100%	FACEREC	1,000,001	1,000,001	26	0.066	0.345	0.112	**0.059**	0.212
	IIG	43,000	43,000	3	0.012	0.137	0.026	**0.008**	0.165
	GEO	500,000	500,000	5	**0.004**	0.111	0.007	0.009	0.300
	TEXT	500	500	24	**0.063**	0.158	0.100	0.232	0.102
	Overall			58	**0.060**	0.256	0.099	0.155	0.172

Table S8: RMSE when extrapolating the correctness κ of robust matching. We report the results for all selected data collections, grouped by corpus, for three sampling fractions $\mu \in [5\%, 10\%, 100\%]$. For each data collection, we measure the empirical correctness from $n^{(0)} = 1$ to $n^{(t)} = n\mu$ records, fit four functional forms, and then report the RMSE between empirical and estimated correctness of n records. The four functional forms include: PYC-MB (Pitman-Yor Correctness functional form, 2 degrees of freedom), ENT (Entropy baseline with no tail complexity, 1 d.o.f), EXP (exponential decay function, 2 d.o.f), POL (polynomial baseline, 2 d.o.f.). We also report results for RND (expected random baseline between 0 and $n^{(t)}$). We report the population size n, the sample size $n^{(t)}$, and the number of data collections c selected from each corpus. We indicate in bold the method with the lowest error rate.

S5 Supplementary Figures S1 to S19

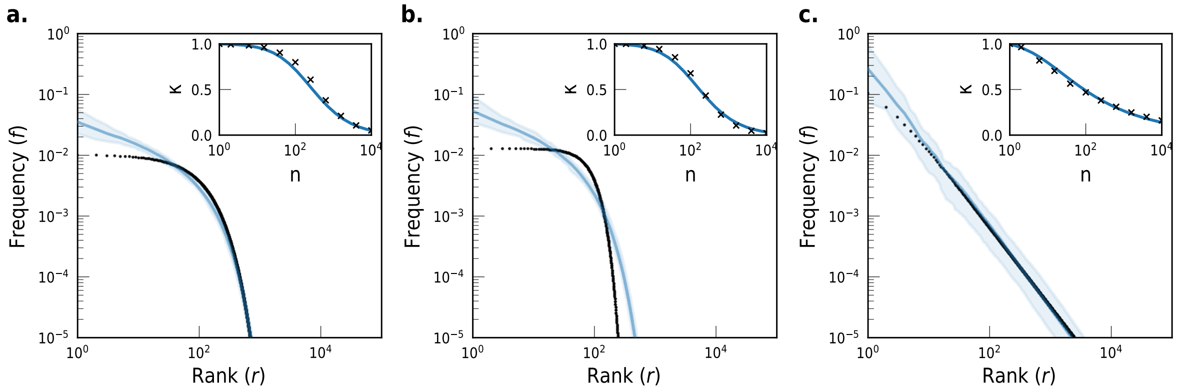

Figure S2: Additional panels supplementing Fig. 1., reporting the fit of the PYC model and the accuracy of the correctness estimator on synthetic data. We report the rank-size distribution of empirical samples (black) and 95% CI sampled from the MAP estimate of the PYP (orange). We sample distributions $X_\phi \sim PY(h^*, \gamma^*)$, using stick-breaking representations, to obtain 95% confidence intervals on the inferred probability mass functions. (Inset) Empirical (black dots) and expected correctness according to the PYC model (solid line) for a population size ranging from 1 to n individuals. (a) Synthetic Geometric corpus (GEOM-1). (b) Synthetic Poisson corpus (POISSON-1). (c) Synthetic Zipf corpus (ZIPF-1).

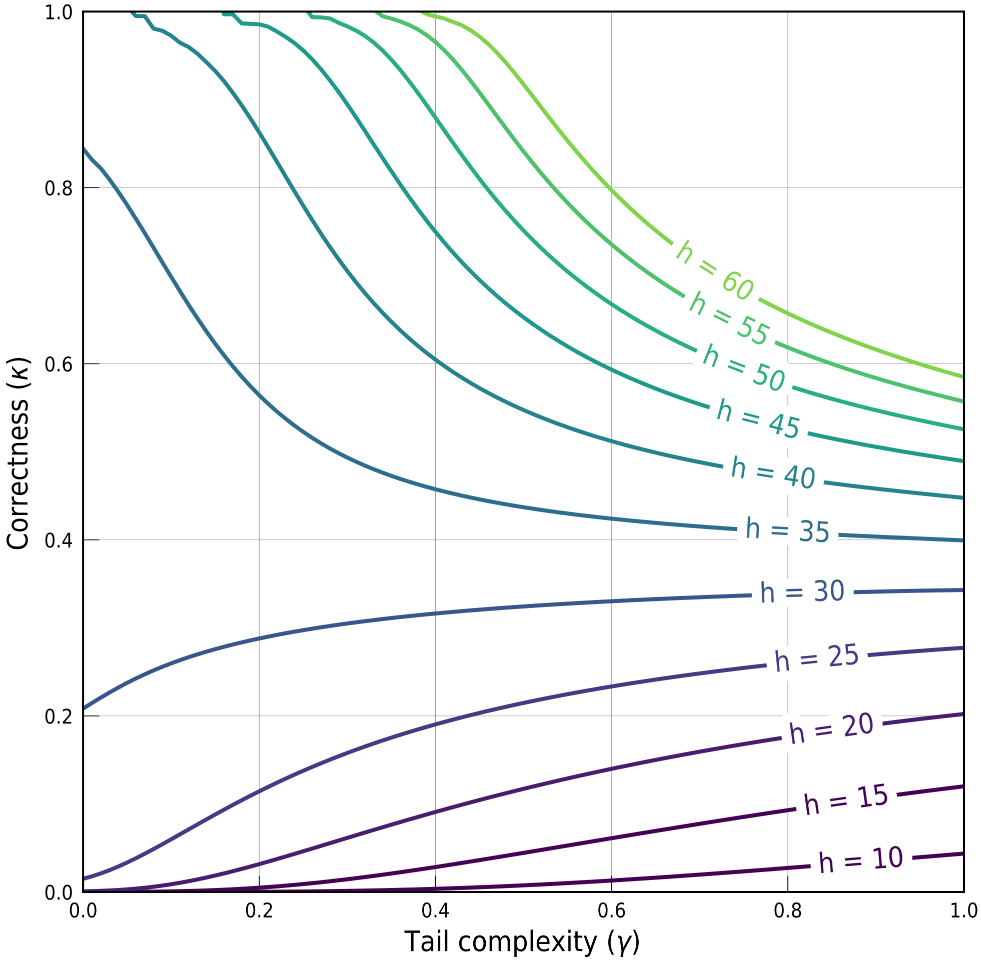

Figure S3: Regimes of correctness. We report the effect of the tail complexity parameter γ on the expected correctness $\mathbb{E}[\kappa \mid h, \gamma]$ for a fixed world population of $n = 7.53$ billion people. Each line represents the correctness for a fixed entropy h from 10 to 60 bits.

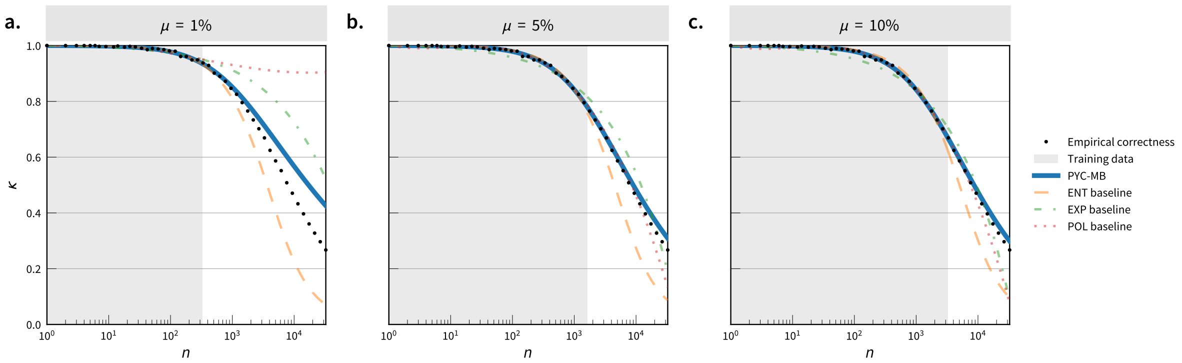

Figure S4: Measurement-based extrapolation of exact matching on the ADULT-1 dataset. We measure the empirical correctness up to (a) $\mu = 1\%$, (b) $\mu = 5\%$, and (c) $\mu = 10\%$ of the original data and, for each sampling fraction μ, train the four functional forms. We display the fitted correctness with solid color lines and the training part with a grey background. We display the empirical correctness from $n = 1$ to $n^{(t)}$ with black dots.

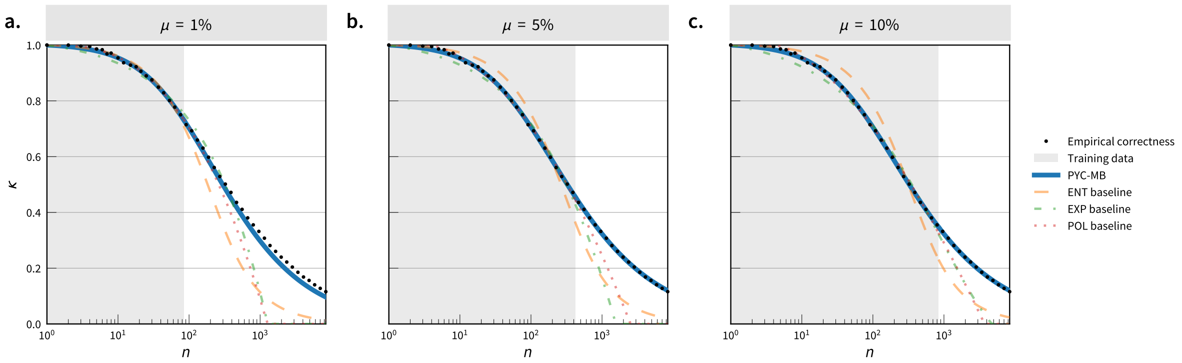

Figure S5: Measurement-based extrapolation of exact matching on the HDV-1 dataset. We measure the empirical correctness up to (a) $\mu = 1\%$, (b) $\mu = 5\%$, and (c) $\mu = 10\%$ of the original data and, for each sampling fraction μ, train the four functional forms. We display the fitted correctness with solid color lines and the training part with a grey background. We display the empirical correctness from $n = 1$ to $n^{(t)}$ with black dots.

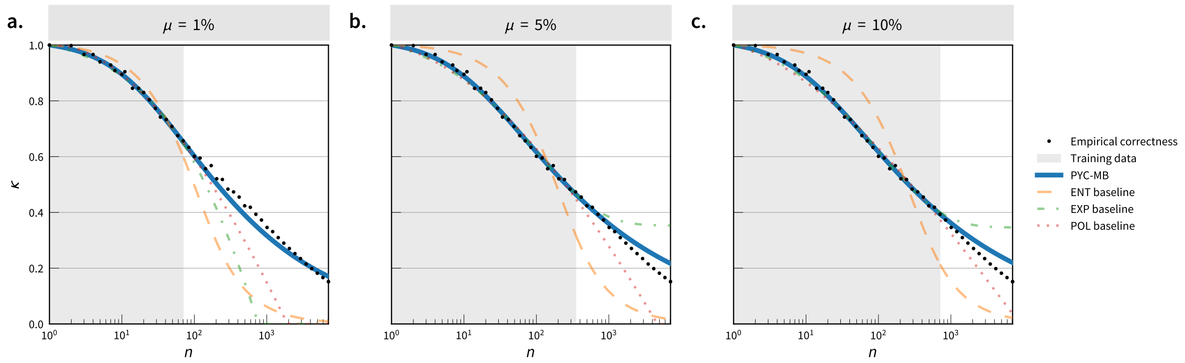

Figure S6: Measurement-based extrapolation of exact matching on the MIDUS-1 dataset. We measure the empirical correctness up to (a) $\mu = 1\%$, (b) $\mu = 5\%$, and (c) $\mu = 10\%$ of the original data and, for each sampling fraction μ, train the four functional forms. We display the fitted correctness with solid color lines and the training part with a grey background. We display the empirical correctness from $n = 1$ to $n^{(t)}$ with black dots.

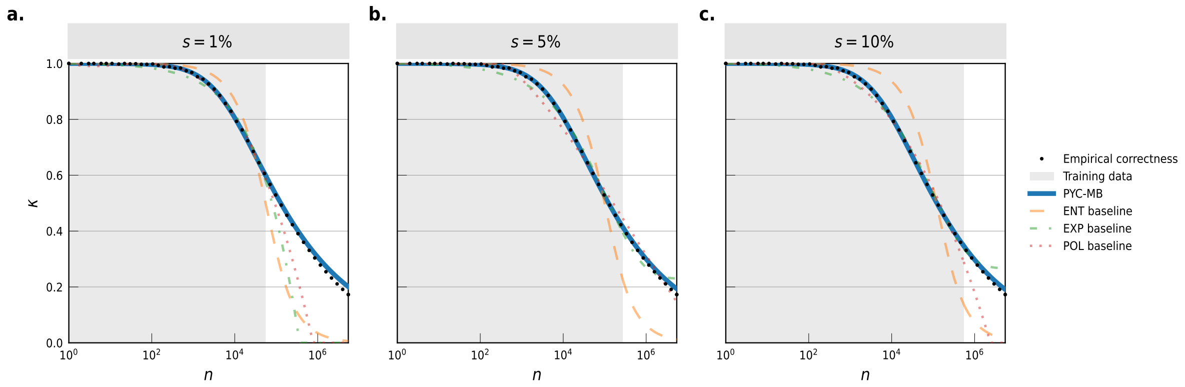

Figure S7: Measurement-based extrapolation of exact matching on the WEB-1 dataset. We measure the empirical correctness up to (a) $\mu = 1\%$, (b) $\mu = 5\%$, and (c) $\mu = 10\%$ of the original data and, for each sampling fraction μ, train the four functional forms. We display the fitted correctness with solid color lines and the training part with a grey background. We display the empirical correctness from $n = 1$ to $n^{(t)}$ with black dots.

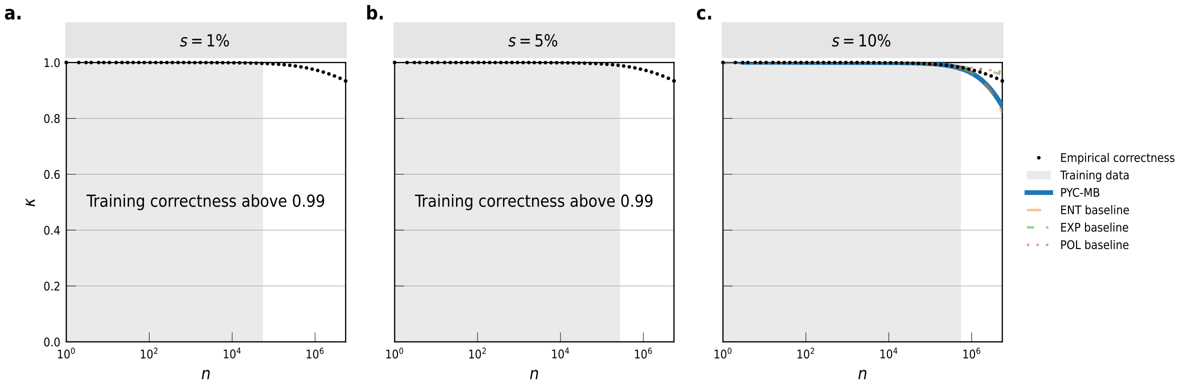

Figure S8: Measurement-based extrapolation of exact matching on the WEB-2 dataset. We measure the empirical correctness up to (a) $\mu = 1\%$, (b) $\mu = 5\%$, and (c) $\mu = 10\%$ of the original data and, for each sampling fraction μ, train the four functional forms. We display the fitted correctness with solid color lines and the training part with a grey background. We display the empirical correctness from $n = 1$ to $n^{(t)}$ with black dots.

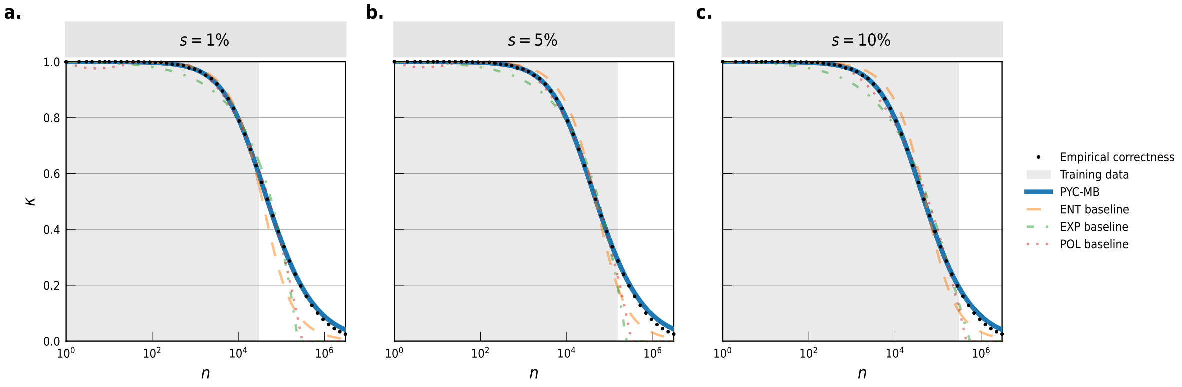

Figure S9: Measurement-based extrapolation of exact matching on the USA-1 dataset. We measure the empirical correctness up to (a) $\mu = 1\%$, (b) $\mu = 5\%$, and (c) $\mu = 10\%$ of the original data and, for each sampling fraction μ, train the four functional forms. We display the fitted correctness with solid color lines and the training part with a grey background. We display the empirical correctness from $n = 1$ to $n^{(t)}$ with black dots.

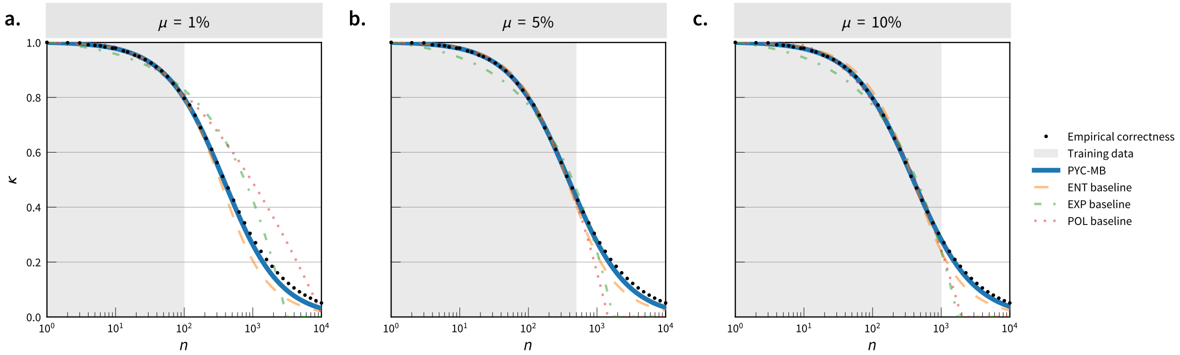

Figure S10: Measurement-based extrapolation of exact matching on the GEOM-1 dataset. We measure the empirical correctness up to (a) $\mu = 1\%$, (b) $\mu = 5\%$, and (c) $\mu = 10\%$ of the original data and, for each sampling fraction μ, train the four functional forms. We display the fitted correctness with solid color lines and the training part with a grey background. We display the empirical correctness from $n = 1$ to $n^{(t)}$ with black dots.

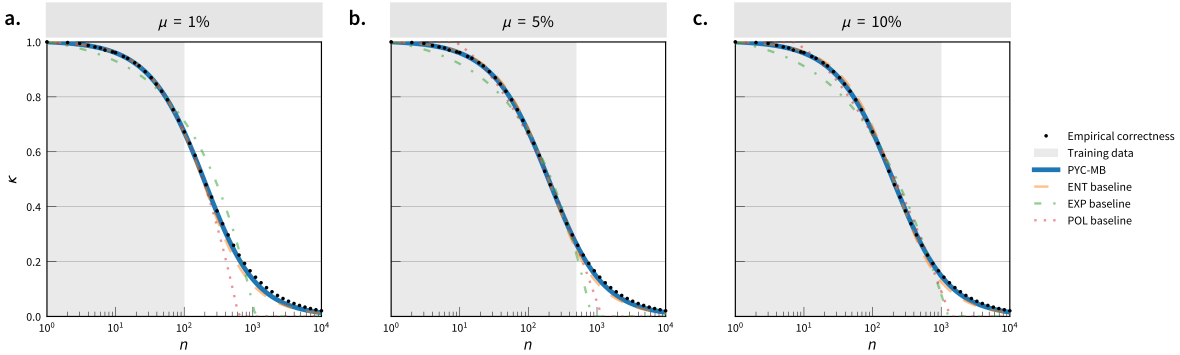

Figure S11: Measurement-based extrapolation of exact matching on the POISSON-1 dataset. We measure the empirical correctness up to (a) $\mu = 1\%$, (b) $\mu = 5\%$, and (c) $\mu = 10\%$ of the original data and, for each sampling fraction μ, train the four functional forms. We display the fitted correctness with solid color lines and the training part with a grey background. We display the empirical correctness from $n = 1$ to $n^{(t)}$ with black dots.

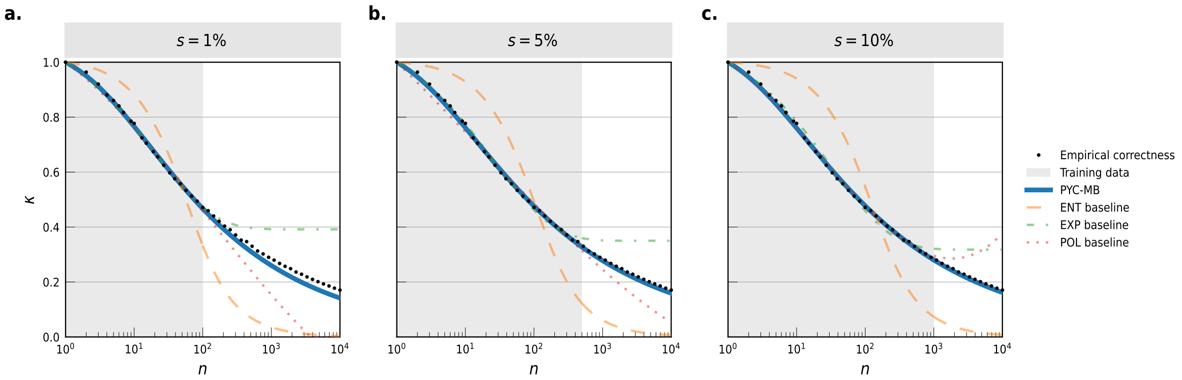

Figure S12: Measurement-based extrapolation of exact matching on the ZIPF-1 dataset. We measure the empirical correctness up to (a) $\mu = 1\%$, (b) $\mu = 5\%$, and (c) $\mu = 10\%$ of the original data and, for each sampling fraction μ, train the four functional forms. We display the fitted correctness with solid color lines and the training part with a grey background. We display the empirical correctness from $n = 1$ to $n^{(t)}$ with black dots.

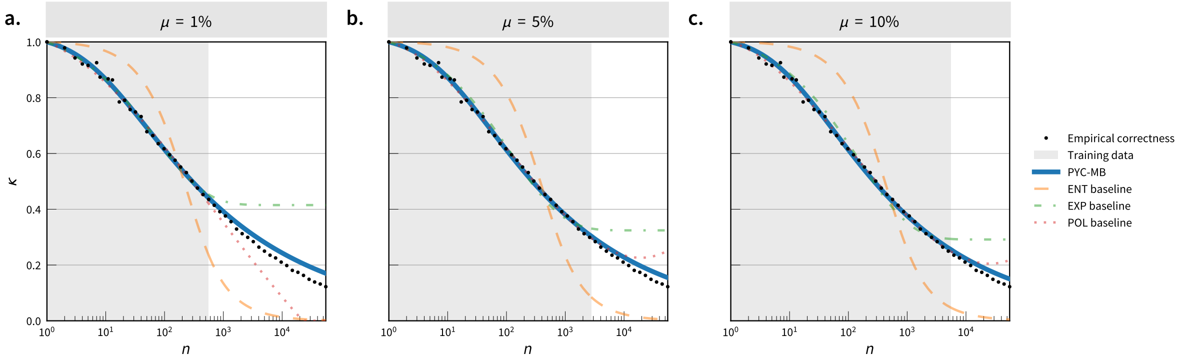

Figure S13: Measurement-based extrapolation of sparse matching on the APPS-1 dataset. We measure the empirical correctness up to (a) $\mu = 1\%$, (b) $\mu = 5\%$, and (c) $\mu = 10\%$ of the original data and, for each sampling fraction μ, train the four functional forms. We display the fitted correctness with solid color lines and the training part with a grey background. We display the empirical correctness from $n = 1$ to $n^{(t)}$ with black dots.

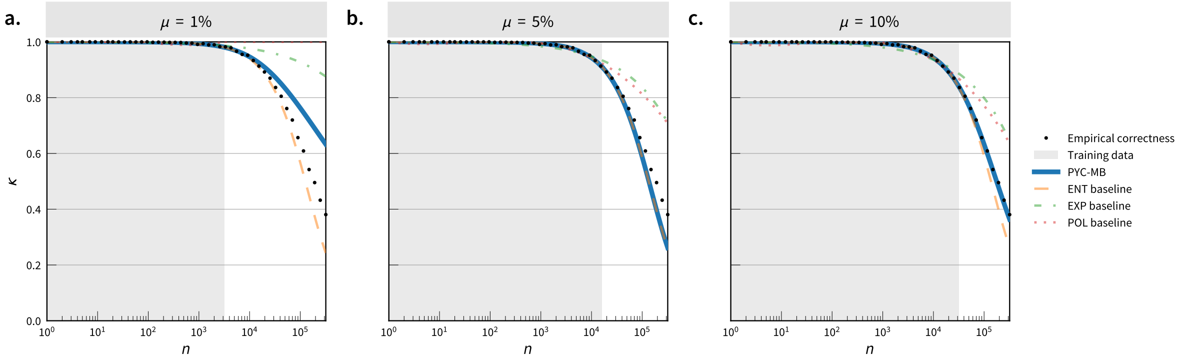

Figure S14: Measurement-based extrapolation of sparse matching on the CALLS-1 dataset. We measure the empirical correctness up to (a) $\mu = 1\%$, (b) $\mu = 5\%$, and (c) $\mu = 10\%$ of the original data and, for each sampling fraction μ, train the four functional forms. We display the fitted correctness with solid color lines and the training part with a grey background. We display the empirical correctness from $n = 1$ to $n^{(t)}$ with black dots.

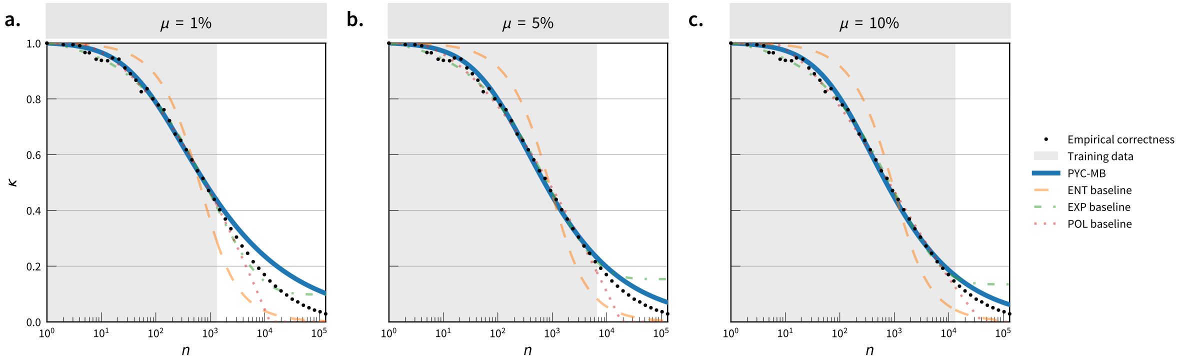

Figure S15: Measurement-based extrapolation of sparse matching on the SHOPS-1 dataset. We measure the empirical correctness up to (a) $\mu = 1\%$, (b) $\mu = 5\%$, and (c) $\mu = 10\%$ of the original data and, for each sampling fraction μ, train the four functional forms. We display the fitted correctness with solid color lines and the training part with a grey background. We display the empirical correctness from $n = 1$ to $n^{(t)}$ with black dots.

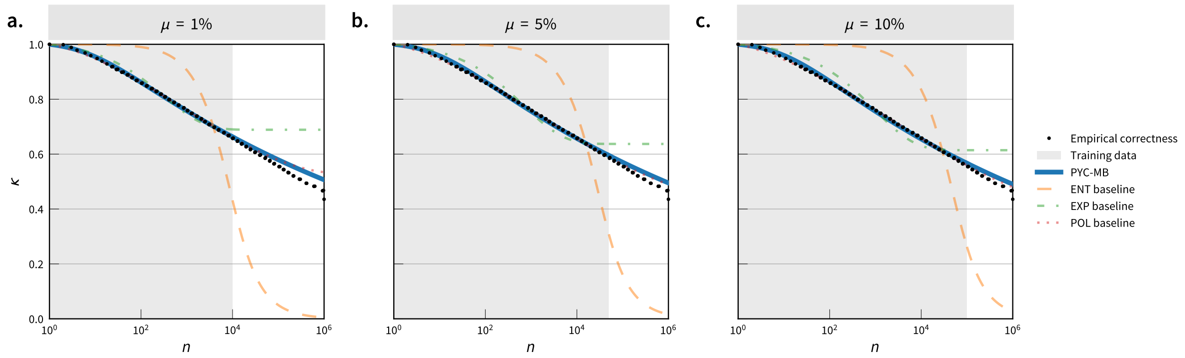

Figure S16: Measurement-based extrapolation of robust matching on the FACEREC-1 dataset. We measure the empirical correctness up to (a) $\mu = 1\%$, (b) $\mu = 5\%$, and (c) $\mu = 10\%$ of the original data and, for each sampling fraction μ, train the four functional forms. We display the fitted correctness with solid color lines and the training part with a grey background. We display the empirical correctness from $n = 1$ to $n^{(t)}$ with black dots.

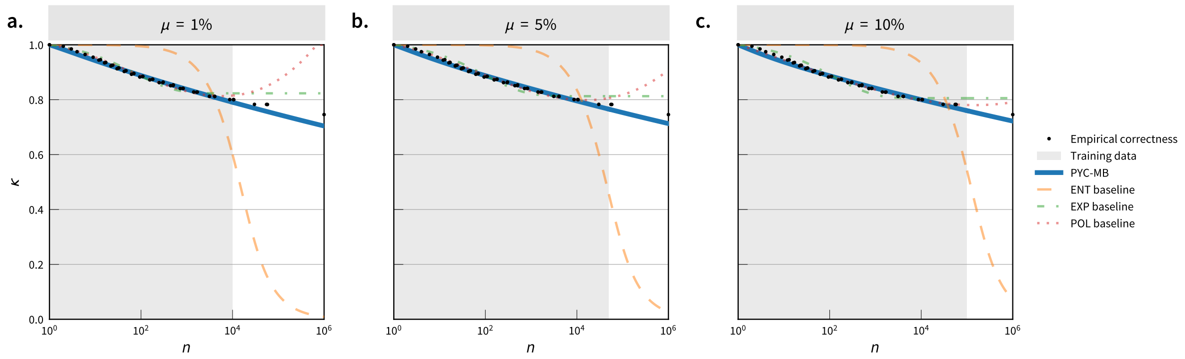

Figure S17: Measurement-based extrapolation of robust matching on the FACEREC-2 dataset. We measure the empirical correctness up to (a) $\mu = 1\%$, (b) $\mu = 5\%$, and (c) $\mu = 10\%$ of the original data and, for each sampling fraction μ, train the four functional forms. We display the fitted correctness with solid color lines and the training part with a grey background. We display the empirical correctness from $n = 1$ to $n^{(t)}$ with black dots.

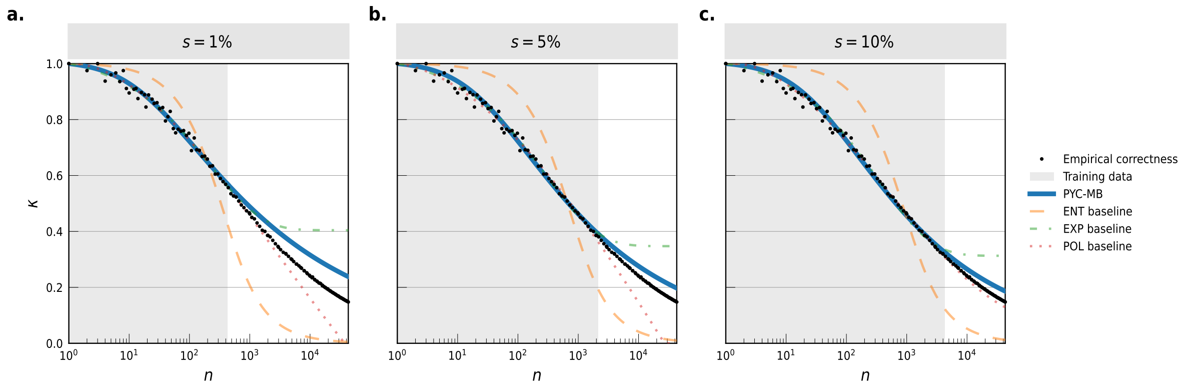

Figure S18: Measurement-based extrapolation of robust matching on the IIG-1 dataset. We measure the empirical correctness up to (a) $\mu = 1\%$, (b) $\mu = 5\%$, and (c) $\mu = 10\%$ of the original data and, for each sampling fraction μ, train the four functional forms. We display the fitted correctness with solid color lines and the training part with a grey background. We display the empirical correctness from $n = 1$ to $n^{(t)}$ with black dots.

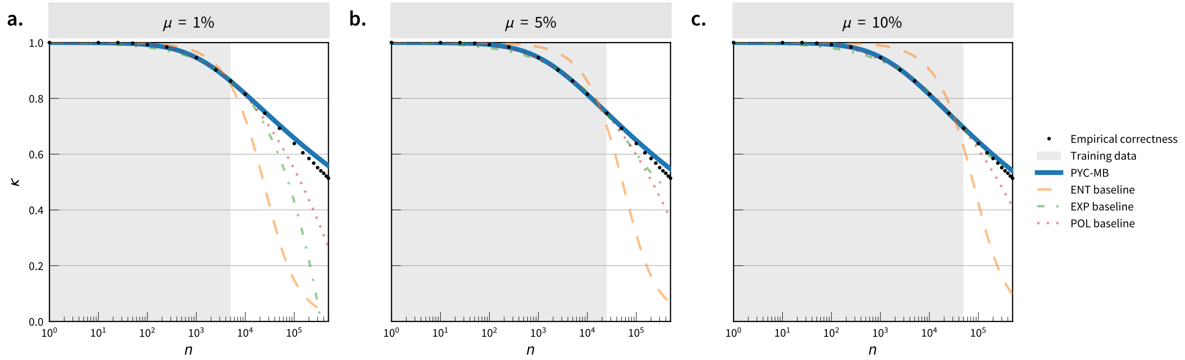

Figure S19: Measurement-based extrapolation of robust matching on the GEO-1 dataset. We measure the empirical correctness up to (a) $\mu = 1\%$, (b) $\mu = 5\%$, and (c) $\mu = 10\%$ of the original data and, for each sampling fraction μ, train the four functional forms. We display the fitted correctness with solid color lines and the training part with a grey background. We display the empirical correctness from $n = 1$ to $n^{(t)}$ with black dots.

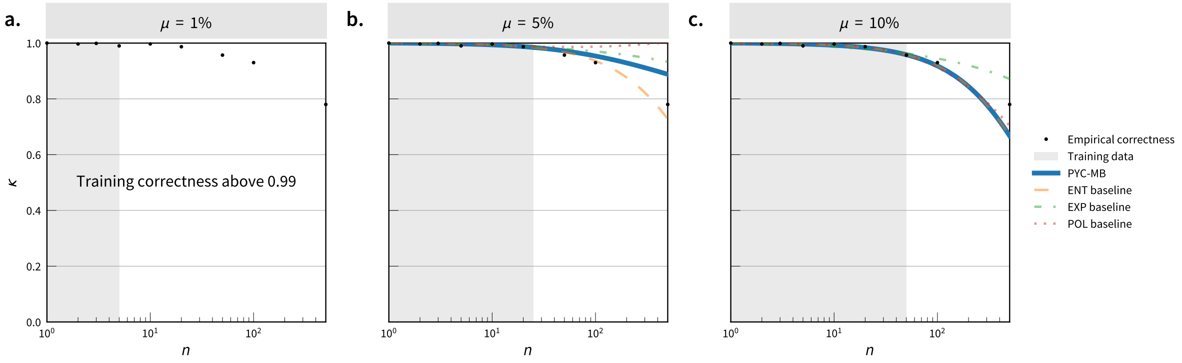

Figure S20: Measurement-based extrapolation of robust matching on the TEXT-1 dataset. We measure the empirical correctness up to (a) $\mu = 1\%$, (b) $\mu = 5\%$, and (c) $\mu = 10\%$ of the original data and, for each sampling fraction μ, train the four functional forms. We display the fitted correctness with solid color lines and the training part with a grey background. We display the empirical correctness from $n = 1$ to $n^{(t)}$ with black dots.

Supplementary References

1. U.S. Census Bureau. *American Community Survey, 2010 American Community Survey 1-Year Estimates* 2010.
2. Dua, D. & Graff, C. *UCI Machine Learning Repository* 2017.
3. INSEE. *Histoire de vie 2003* Apr. 26, 2006.
4. Brim, O. G. *et al. Midlife in the United States (MIDUS 1), 1995-1996* 2020.
5. Eckersley, P. How unique is your web browser? *Proc. 10th Int. Priv. Enh. Technol.* **2010,** 1–18 (1 July 21, 2010).
6. Budington, B. *Panopticlick: Fingerprinting Your Web Presence* San Francisco, CA, Jan. 2016.
7. Farzanehfar, A., Houssiau, F. & de Montjoye, Y.-A. The risk of re-identification remains high even in country-scale location datasets. *Patterns* **2,** 100204 (3 Mar. 12, 2021).
8. Oliner, A. J., Iyer, A. P., Stoica, I., Lagerspetz, E. & Tarkoma, S. Carat: collaborative energy diagnosis for mobile devices. *Proc. ACM Conf. Embedded Netw. Sensor Syst.* **11,** 1–14 (Nov. 11, 2013).
9. *The Instacart Online Grocery Shopping Dataset 2017* https://www.instacart.com/datasets/grocery-shopping-2017 (2020).
10. Kemelmacher-Shlizerman, I., Seitz, S. M., Miller, D. & Brossard, E. The MegaFace Benchmark: 1 Million Faces for Recognition at Scale. *Proc. IEEE Comput. Soc. Conf. Comput. Vis. Pattern Recognit.* **2016,** 4873–4882 (June 30, 2016).
11. Tournier, A. J. & de Montjoye, Y.-A. Expanding the attack surface: Robust profiling attacks threaten the privacy of sparse behavioral data. *Sci. Adv.* **8,** eabl6464 (33 2022).
12. Creţu, A.-M. *et al.* Interaction data are identifiable even across long periods of time. *Nat. Commun.* **13,** 313 (1 Jan. 25, 2022).
13. Saedi, C. & Dras, M. Siamese networks for large-scale author identification. *Comput. Speech Lang.* **70,** 101241 (Nov. 1, 2021).
14. Rocher, L., Hendrickx, J. M. & de Montjoye, Y.-A. Estimating the success of re-identifications in incomplete datasets using generative models. *Nat. Commun.* **10** (3069 July 23, 2019).
15. Archer, E., Park, I. M. & Pillow, J. W. Bayesian Entropy Estimation for Countable Discrete Distributions. *J. Mach. Learn. Res.* **15,** 2833–2868 (2014).
16. Paninski, L. Estimation of entropy and mutual information. *Neural Comput.* **15,** 1191–1253 (6 June 1, 2003).
17. Achara, J. P., Acs, G. & Castelluccia, C. On the Unicity of Smartphone Applications. *Proc. ACM Workshop Priv. Electron. Soc.* **2014,** 27–36 (2015).

18. Sekara, V., Alessandretti, L., Mones, E. & Jonsson, H. Temporal and cultural limits of privacy in smartphone app usage. *Sci. Rep.* **11,** 3861 (1 Feb. 16, 2021).

19. Grother, P., Ngan, M. & Hanaoka, K. *Face Recognition Vendor Test (FRVT) Part 2: Identification* research rep. NISTIR 8271 (NIST, Sept. 2019). 186 pp.

20. Friedman, L. *et al.* Biometric Performance as a Function of Gallery Size. *Appl. Sci.* **12,** 11144 (21 Nov. 3, 2022).

21. Baveja, M., Yuan, H. & Wein, L. M. Asymptotic Biometric Analysis for Large Gallery Sizes. *IEEE Trans. Inf. Forensics Secur.* **5,** 955–964 (4 Dec. 2010).
